# Entomological impact of mass administration of ivermectin and dihydroartemisinin-piperaquine in The Gambia: a cluster-randomized controlled trial

**DOI:** 10.1186/s13071-022-05557-4

**Published:** 2022-11-17

**Authors:** Harouna M. Soumare, Edgard Diniba Dabira, Muhammed M. Camara, Lamin Jadama, Pa Modou Gaye, Sainey Kanteh, Ebrima A. Jawara, Amie Kolleh Njie, Fatou Sanneh, Mamadou Ousman Ndiath, Steven W. Lindsay, Bakary Conteh, Sainey Ceesay, Nuredin Mohammed, Michael Ooko, John Bradley, Chris Drakeley, Annette Erhart, Teun Bousema, Umberto D’Alessandro

**Affiliations:** 1 Medical Research Council Unit The Gambia at the London, School of Hygiene and Tropical Medicine, Banjul, The Gambia; 2 Department of Biosciences, Durham University, Durham, UK; 3 Faculty of Infectious & Tropical Diseases, The London School of Hygiene and Tropical Medicine, London, UK; 4 Radboud Institute for Health Sciences, Radboud University Medical Center, Nijmegen, The Netherlands; 5 MRC International Statistics and Epidemiology Group, London School of Hygiene and Tropical Medicine, London, UK

**Keywords:** Ivermectin, Mass Drug Administration, Malaria, Vector

## Abstract

**Background:**

Vector control interventions in sub-Saharan Africa rely on insecticide-treated nets and indoor residual spraying. Insecticide resistance, poor coverage of interventions, poor quality nets and changes in vector behavior threaten the effectiveness of these interventions and, consequently, alternative tools are needed. Mosquitoes die after feeding on humans or animals treated with ivermectin (IVM). Mass drug administration (MDA) with IVM could reduce vector survival and decrease malaria transmission. The entomological impact of MDA of combined IVM and dihydroartemisinin-piperaquine was assessed in a community-based, cluster-randomized trial.

**Methods:**

A cluster-randomized trial was implemented in 2018 and 2019 in 32 villages in the Upper River Region, The Gambia. The with the inhabitants of 16 intervention villages eligible to receive three monthly rounds of MDA at the beginning of the malaria transmission season. Entomological surveillance with light traps and human landing catches (HLC) was carried out during a 7- to 14-day period after each round of MDA, and then monthly until the end of the year. The mosquitocidal effect of IVM was determined by direct membrane feeding assays.

**Results:**

Of the 15,017 mosquitoes collected during the study period, 99.65% (*n* = 14,965) were *Anopheles gambiae* sensu lato (*An. gambiae* s.l.), comprising *Anopheles arabiensis* (56.2%), *Anopheles coluzzii* (24.5%), *Anopheles gambiae* sensu stricto (*An. gembiae* s.s.; 16.0%) and *Anopheles funestus* sensu lato (*An. funestus* s.l.; 0.35%). No effect of the intervention on vector parity was observed. Vector density determined on light trap collections was significantly lower in the intervention villages in 2019 (adjusted incidence rate ratio: 0.39; 95% confidence interval [CI]: 0.20, 0.74; *P* = 0.005) but not in 2018. However, vector density determined in HLC collections was similar in both the intervention and control villages. The entomological inoculation rate was significantly lower in the intervention villages than in the control villages (odds ratio: 0.36, 95% CI: 0.19, 0.70; *P*  = 0·003). Mosquito mortality was significantly higher when blood fed on IVM-treated individuals up to 21 days post-treatment, particularly in adults and individuals with a higher body mass index.

**Conclusion:**

Mass drug administration with IVM decreased vector density and the entomological inoculation rate while the effect on vector parity was less clear. Survival of mosquitoes fed on blood collected from IVM-treated individuals was significantly lower than that in mosquitoes which fed on controls. The influence of host characteristics on mosquito survivorship indicated that dose optimization could improve IVM efficacy. Future detailed entomological evaluation trials in which IVM is administered as stand-alone intervention may elucidate the contribution of this drug to the observed reduction in transmission.

**Graphical Abstract:**

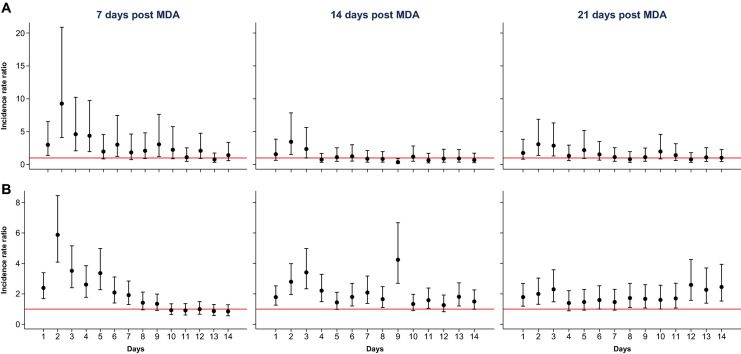

**Supplementary Information:**

The online version contains supplementary material available at 10.1186/s13071-022-05557-4.

## Introduction

Vector control interventions such as long-lasting insecticidal nets (LLINs) and indoor residual spraying (IRS) are the main components of malaria vector control in sub-Saharan Africa [1, 2]. In The Gambia, prompt diagnosis and treatment with artemisinin-based combinations and the large-scale deployment of LLINs and IRS have resulted in a substantial decline of the malaria burden [3, 4]. Nevertheless, malaria transmission, which is highly seasonal, has not been stopped completely. Significant resistance to dichlorodiphenyltrichloroethane (DDT) and pyrethroids has been recently reported [5–7], which may partly explain, in addition to climate change [8], changes in the density distribution and biting and resting behaviors of *Anopheles gambiae* sensu lato (*An. gambiae* s.l.), the dominant malaria vector, and the heterogeneity of malaria transmission [9, 10]. Indeed, LLINs and IRS protect against those vectors which bite and rest indoors [11], but changes in vector behaviors, such as outdoor biting and/or biting earlier [10, 12], vector biodiversity and environmental change [13], may decrease the protection provided by these interventions. A recent study in The Gambia reported a significant preference of *Anopheles arabiensis* for outdoor resting [14], thereby decreasing the effect of standard vector control interventions such as IRS and LLINs. This behavior highlights the need for insecticides other than pyrethroids [1] and for the targeting of vectors currently able to escape standard control interventions [15].

Ivermectin (IVM) is an endectocide, broad-spectrum systemic drug that is efficacious against nematodes and arthropods [16]. When IVM is ingested through a blood meal from an IVM-treated human or animal, it exerts a lethal effect by acting on the glutamate-gated chloride channels of arthropods [17, 18], disrupting their neuromuscular transmission and leading to paralysis and death [19]. IVM has the potential to target both insecticide-resistant and outdoor-biting* Anopheles* mosquitoes [2, 12]. Therefore, mass drug administration (MDA) of IVM may decrease the survival of human biting mosquitoes, regardless of their behavior [20–23] and insecticide resistance status [24, 25]. In Africa, MDA with IVM was found to temporarily alter the age structure of mosquito populations and reduce malaria transmission by reducing vector survival and thus the capacity to complete the malaria parasite sporogonic cycle [26, 27]. The effect of MDA with IVM on malaria transmission can be assessed by implementing a community-based, cluster-randomized trial [28]. In Burkina Faso, repeated rounds of MDA with IVM reduced the incidence of clinical malaria, without concurrent reductions in entomological exposure indicators[29], highlighting the need for dedicated entomological evaluations on the impact of IVM on mosquito populations. Here, we report a detailed analysis of the entomological impact of MDA with IVM and dihydroartemisinin-piperaquine within a cluster-randomized trial that was carried out in eastern Gambia [30].

## Methods

### Study site and trial procedures

Thirty-two villages which were located at least 3 km apart were selected according to malaria prevalence determined by an earlier cross-sectional survey [31] and randomized to either the intervention (16 villages) or control group (16 villages). A buffer zone of 2-km radius was established around each intervention village to minimize contamination from neighboring untreated villages [30]. All villages within the buffer zone received the intervention although they were not included in the evaluation. In 2018 and 2019, monthly rounds of MDA with IVM (Laboratorio Elea, Los Polvorines, Argentina), administered at a dose of 300–400 μg/kg body weight per day for 3 consecutive days, and dihydroartemisinin-piperaquine (DP; Guilin Pharmaceuticals, Guilin, Guangxi, China), administered according to body weight following the manufacturer’s guidelines, were performed for three consecutive months at the beginning of each malaria transmission season: in August, September and October of 2018 and in July, August and September of 2019. All drugs were administered orally. During each round of MDA, the research team covered all 16 intervention villages between 12 and 14 days, with each individual village covered within a period of about 3–5 days.

Malaria transmission in The Gambia is highly seasonal, with a peak in October–November [32]. Information on rainfall, temperature and humidity are presented in Additional file 1: Table S1. The annual temperature and humidity were similar for both study years.

### Entomological collections

Mosquitoes were collected using standard US Center for Disease Control and Prevention (CDC) light traps (CDC-LT; CDC, Atlanta, GA, USA) hung from the ceiling at the foot end of a bed with the light 70–150 cm above the ground [33]. Intensive sampling to measure mosquito parity and density was carried out from 7 to 14 days after each MDA round, and then monthly until the end of the transmission season (December). Mosquitoes were collected over three consecutive nights in six randomly selected houses per village in all intervention villages and in six randomly selected houses in eight control villages. For the other control villages, collections were carried out at the same time for one night. CDC-LT were set up by trained field assistants and run for 12 h, from 19:00 h until 7:00 h. The CDC-LT were checked every 4 h.

Monthly human landing catches (HLC), both indoors and outdoors, were carried out in four intervention and four control villages that were randomly selected, both in 2018 and in 2019. In 2018, collections were done for three consecutive nights in six houses randomly selected using village census identification; in 2019, collections were done in three houses per village for two consecutive nights. HLC were done from 19:00 h to 07:00 h by four volunteers (2 indoors and 2 outdoors) who rotated every 2 h to avoid collector bias.

Each morning, all collected mosquitoes were transported to the laboratory where they were morphologically identified and stored in separate tubes with silica gel for further analysis, while other anophelines and culicine mosquitoes were counted and then discarded. A subset of *An. gambiae* s.l. (*N* = 12 per night from HLC [6 outdoors and 6 indoors] and* N* = 10 per room per day from CDC-LT) were used to estimate mosquito parity [34]. Head and thorax (500 per each collection round per arm if available) were used for the detection of *Plasmodium falciparum* circumsporozoite protein (CSP) by enzyme-linked immunosorbent assay (ELISA) [35]. Abdomens from a subset of samples processed for ELISA were used for mosquito identification by molecular methods [36].

### Membrane feeding experiment

A subset of the study population, comprising 80 randomly selected participants (50% aged 4–10 years old [children] and 50% aged ≥ 18 years [adults]) living in one intervention village (*N* = 40) and one control village (*N* = 40), were selected for participation in the direct membrane feeding assay (DMFA). These villages were chosen for their proximity to the insectary of the Medical Research Council Unit The Gambia (MRCG) field station in Basse, The Gambia. Venous blood samples were collected in 4-ml tubes coated in lithium-heparin (BD, Franklin Lakes, NJ, USA) at 7, 14 and 21 days after the administration of the first dose of IVM from all participants in the intervention villages and in the control villages. For the intervention villages, in 2018, study participants were randomly selected, without confirming whether they had actually taken IVM; in 2019, only individuals who had taken all IVM doses under direct supervision were included in the analysis.

*Anopheles coluzzii* mosquitoes were reared in the insectary at 27 °C and approximately 70-80% relative humidity (RH) under a 12/12-h day/night cycle and fed on 5–10% glucose. Immediately after phlebotomy, two aliquots of 400–500 μl of whole blood were dispensed into two glass feeders, and 50 female *An. coluzzii* mosquitoes (aged 2–6 days) per feeder (total* N* = 100) were allowed to feed through a Parafilm membrane for 20 min. After feeding, partially fed mosquitoes were removed and fully fed mosquitoes were kept at 27 °C in a specific container. Mortality was estimated daily, up to 14 days post-feeding.

### Entomological parameters and statistical analysis

Vector density, parity rate, species composition and sporozoite rate were determined from the CDC-LT collections and HLC. The biting rate and the entomological inoculation rate (EIR) were determined only from HLC collections.

Mosquito density was calculated as the number of collected mosquitoes divided by the number of trapping nights. For CDC-LT, mixed-effects generalized models with negative binomial distribution were used to determine the impact of MDA on mosquito density, controlling for MDA round as a categorical fixed effect. The village was included in the model as a random effect. Since the number of villages for HLC collections was small, the effect of the intervention was estimated based on village-level summaries [37]. The analysis was conducted in two stages: first, we used a Poisson regression model to compute a residual for each village after adjusting for the MDA round; second, we calculated the ratio of the observed to the predicted events for each village. The unpaired t-test to determine the mean difference between the two groups.

The parity rate was estimated for each collection method by dividing the number of parous mosquitoes by the total number of parous and nulliparous mosquitoes collected. For CDC-LT, mixed-effects logistic regression was used to model the impact of MDA on parity, with village included as a random effect. The models were adjusted for MDA round. For HLC collections, the effect of the intervention was based on village-level summaries. The first step was to fit a logistic regression model that adjusted for the MDA round on individual-level data, ignoring the intervention effect and clustering. Next, we estimated the expected number of parous mosquitoes for each village and calculated residuals as a ratio of the number of expected parous mosquitoes to the number of observed parous mosquitoes. The intervention effect was calculated as the ratio of mean residuals between treatment arms. To determine the significance of the difference between the treatment arms, the unpaired t-test was applied to the village-level residuals.

Sporozoite rate was estimated by dividing the number of CSP-positive mosquitoes by the total number of mosquitoes analyzed. We used mixed-effects logistic regression to model the impact of MDA on sporozoite rate with village included as a random effect.

The biting rate was estimated by dividing the number of mosquitoes that were collected by the number of those capturing the mosquitoes. The effect of the intervention on the biting rate was estimated based on village-level summaries. The analysis was conducted in two stages: first, we used a Poisson regression model to compute a residual for each village after adjusting for the MDA round; second, we calculated the ratio of the observed to the predicted events for each village. The unpaired t-test was used to determine the significance of the mean difference between the two groups. Permutation tests were used to validate t-test* P*-values.

A summary EIR for the two transmission seasons (2018 and 2019) was estimated by multiplying the sporozoite rate for the HLC by the biting rate, multiplied by 360 days. A mixed-effects generalized model with negative binomial distribution was fitted to determine the impact of IVM on mosquito survival following DFMA. Study subject was included in the model as a random effect. The model was adjusted for age, gender and body mass index (BMI) of the study subject. The analysis was further stratified by year of MDA administration and by age and gender of the study participant.

### Ethical considerations

The study protocol received ethical approval from The Gambia Government/MRC Joint Ethics Committee and the London School of Hygiene and Tropical Medicine Ethics Committee. Before any procedure was initiated, written informed consent was provided by adult participants and by parents/guardians of children. Children aged 12 to  < 18 years provided their assent.

## Results

Between August 2018 and January 2019 and between July and December 2019, a total of 4116 trapping nights involving CDC light-traps in 192 households and 924 trapping nights involving HLC in 72 households were completed. When the results of both trapping methods were combined, most vectors (99.6%, 14,964/15,017 mosquitoes) were morphologically identified as *Anopheles gambiae* s.l., with no difference between study arms (Table 1). The remaining mosquitoes were identified as *Anopheles funestus* sensu lato (*An. funestus* s.l.)

**Table 1:** Vector species composition by study arm and combined study years (2018 and 2019)

Vector species identification	Intervention, % (*N*)	Control, % (*N*)	Total, % (*N*)
*Morphological identification*			
*Anopheles gambiae* sensu lato	99.4 (5407)	99.8 (9557)	14,964 (99.65)
*Anopheles funestus* sensu lato	0.6 (30)	0.24 (23)	0.4 (53)
Total *N*	5437	9580	15,017
*Molecular identification*^a^			
*Anopheles arabiensis*	59.5 (1192)	53.2 (1166)	56.2 (2,358)
*Anopheles coluzzii*	26.1 (524)	23.0 (504)	24.5 (1,028)
*Anopheles gambiae* sensu stricto	11.9 (238)	19.7 (432)	16.0 (670)
*Anopheles gambiae/coluzzii*	50 (2.5)	4.1 (91)	3.3 (141)
Total *N*	2004	2,193	4197

*N* Number of mosquitoes

^a^Only* Anopheles gambiae* sensu lato

During the 2 years of the study, the mean number of *An. gambiae* s.l. caught per night by CDC-LT was 1.90 and 0.88 in the control and intervention arms, respectively, and by HLC, 5.46 and 3.44 in the control and intervention arms, respectively (Table 2).

**Table 2:** Mean density of *Anopheles gambiae* sensu lato per trapping night by year and collection method

Year	Collection method	Arm	Number of mosquitoes collected	Trapping nights (*N*)	Mean density per trapping night
2018	CDC-LT	Control	1571	1002	0.81
2018	CDC-LT	Intervention	564	858	0.26
2018	HLC	Control	4583	360	12.49
2018	HLC	Intervention	2358	324	3.14
2019	CDC-LT	Control	3088	912	2.45
2019	CDC-LT	Intervention	1914	1344	1.04
2019	HLC	Control	315	120	2.35
2019	HLC	Intervention	571	120	4.56
2018 + 2019	LTC	Control	4659	1914	1.90
2018 + 2019	LTC	Intervention	2478	2202	0.88
2018 + 2019	HLC	Control	4898	480	5.46
2018 + 2019	HLC	Intervention	2929	444	3.44

*CDC-LTC* US Center for Disease Control and Prevention light trap, *HLC* human landing catch

Species identification by PCR (molecular method) was performed on 4197 *An. gambiae* s.l. samples. Of these, approximately one half (56.2%,* N* = 2358) were identified as *An. arabiensis*, 24.5% (1028) as *An. coluzzii* and 16.0% (670) as *Anopheles gambiae* sensu stricto (*An. gambiae* s.s., with no difference between intervention and control villages (Table 1).

Parity could be successfully assessed in 1169 mosquitoes captured by CDC-LT, representing about 17% (1169/6921) of all trapped mosquitoes, with the others being often desiccated prior to emptying CDC light-traps. The proportion of mosquitoes on which parity assessment could be performed was similar between the intervention (22.6%; 458/2029) and control (14.5%; 711/4892) villages, without any indication of bias. There was no difference in parity rates as estimated from CDC-LT collections between the intervention and control arms in both 2018 (adjusted relative risk [RR]: 1.17; 95% CI: 0.93, 1.47; *P *= 0.18) and 2019 (adjusted RR: 1.01; 95% confidence interval [CI]: 0.95, 1.07; *P* = 0.71) (Table 3). Parity as determined by HLC tended to be lower in the intervention group than in the control group, both in 2018 (adjusted RR: 0.87; 95% CI: 0.75, 1.01; *P* = 0.055) and 2019 (adjusted RR: 0.93; 95% CI: 0.65,1.34; *P* = 0.649).

**Table 3:** Vector parity and density by year and study group

Parameter	Methods	2018				2019			
		Control, % (*n*/*N*)	Intervention, % (*n*/*N*)	Adj. RR (95% CI)	*P*-value	Control, % (*n*/*N*)	Intervention, % (*n*/*N*)	Adj. RR (95% CI)	*P*-value
Parity	CDC-LT	53.2 (91/171)	54.9 (50/91)	1.17 (0.93, 1.47)	0.181	81.7 (441/540)	84.2 (309/367)	1.01 (0.95, 1.52)	0.708
	HLC	68.4 (807/1180)	61.5 (475/772)	0.87 (0.75, 1.01)	0.055	89.5 (111/124)	76.3 (132/173)	0.93 (0.65, 1.34)	0.649
Density^a^	CDC-LT	282.2 (1571/1002)	118.3 (564/858)	0.47 (0.14, 1.57)	0.213	609.5 (3088/912)	256.3 (1914/1344)	0.39 (0.20, 0.74)	0.005
	HLC	2291.5 (4583/360)	1310.0 (2358/324)	0.45 (0.15, 1.35)	0.125	472.5 (315/120)	856.5 (571/120)	1.94 (0.88, 4.28)	0.088

*Adj. IRR* adjusted incidence rate ratio,* CI* confidence interval, *n* number of parous mosquitoes, *N* number of dissected mosquitoes, OR odds ratio,* RR* relative risk

^a^Number of mosquitoes per trapping nights multiplied by 180 days (30 days per month multiplied by 6 months per season per year)

Vector density estimated from CDC-LT collections was lower in the intervention villages than in the control villages, particularly in 2019 (adjusted RR: 0.39; 95% CI: 0.20, 0.74; *P* = 0.005), but in 2018 this difference did not reach statistical significance (Table 3). However, vector density as estimated by HLC tended to be lower in 2018 and higher in 2019 in the intervention villages compared to the control villages (Table 3).

Using HLC data, no statistically significant differences in biting and sporozoite rates were observed between the intervention and control groups (Table 4). However, the overall EIR was significantly lower in the intervention group than in the control group (odds ratio [OR]: 0.36; 95% CI: 0.19, 0.70; *P* = 0.003).

**Table 4:** Vector sporozoite rate, biting rate and entomological inoculation rate for 2018 and 2019 combined

Parameter	Methods	Control, % (*n*/*N*)	Intervention, % (*n*/*N*)	Adj, IRR (95% CI)	*P*-value
Biting rate, %	HLC	3.4 (4898/1440)	2.2 (2929/1344)	0.78 (0.23, 2.77)	0.701

Parameter	Methods	Control, % (*n*/*N*)	Intervention, % (*n*/*N*)	Crude OR (95% CI)	*P*-value
Sporozoite rate, %	CDC-LT and HLC	1.1 (68/6001)	0.8 (31/3939)	0.69 (0.46, 1.06)	0.091
EIR	HLC	56.9 (4898/360) × (29/2498) × 360	20.8 (2929/360) × (13/1835) × 360	0.36 (0.19, 0.70)	0.003

* EIR* Entomological inoculation rate

Biting rate: n number of mosquito collected; N number of capturers

Sporozoite rate: n number of mosquito positive for sporozoite; N number of mosquito processed for sporozoite

**Fig. 1:**
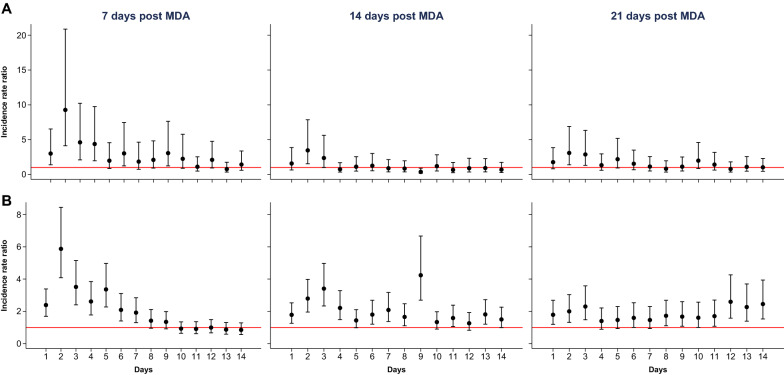
Incidence rate ratio (IRR) and 95% confidence interval of mosquito mortality for the intervention group versus the control group over time post-intervention. The red line represents an IRR of 1. Results for 2018 (**a**) and 2019 (**b**). Analysis was adjusted for age, gender and body mass index of the study subject. MDA, Mass drug administration

The mortality of mosquitoes fed on blood collected from individuals in the intervention villages was significantly higher than that of those fed on blood collected from individuals in the control villages. The highest mortality was observed during the first days after blood-feeding (Fig. 1), particularly in mosquitoes fed on blood collected from individuals 7 and 14 days after the first IVM dose. Mortality remained high among mosquitoes fed on blood collected from individuals 21 days after the first IVM dose (Fig. 1; Table 5).

**Table 5:** Incidence rate ratios for the effects of mass drug administration on mosquito mortality based on combined 2018 and 2019 data

Variables	7 days post-MDA			14 days post-MDA			21 days post-MDA		
	IRR	95% CI	*P*-value	IRR	95% CI	*P*-value	IRR	95% CI	*P-*value
*Treatment day*									
1	2.38	1.72, 3.29	< 0.001	1.64	1.16, 2.31	0.005	1.69	1.16, 2.45	0.006
2	6.20	4.42, 8.69	< 0.001	2.87	2.03, 4.07	< 0.001	2.04	1.39, 3.00	< 0.001
3	3.81	2.69, 5.40	< 0.001	3.14	2.18, 4.53	< 0.001	2.34	1.57, 3.49	< 0.001
4	3.02	2.12, 4.29	< 0.001	1.64	1.13, 2.37	0.009	1.33	0.88, 2.01	0.171
5	2.91	2.03, 4.17	< 0.001	1.35	0.93, 1.94	0.113	1.52	1.00, 2.29	0.048
6	2.13	1.48, 3.08	< 0.001	1.64	1.12, 2.41	0.012	1.51	1.00, 2.30	0.051
7	1.82	1.26, 2.63	0.001	1.67	1.13, 2.48	0.010	1.33	0.88, 1.99	0.176
8	1.53	1.06, 2.20	0.023	1.42	0.97, 2.07	0.072	1.46	0.97, 2.19	0.069
9	1.44	1.01, 2.06	0.046	2.18	1.47, 3.24	< 0.001	1.48	0.99, 2.21	0.055
10	0.99	0.70, 1.40	0.959	1.33	0.91, 1.93	0.143	1.64	1.07, 2.51	0.022
11	0.97	0.67, 1.39	0.848	1.33	0.90, 1.98	0.154	1.55	1.03, 2.35	0.037
12	1.23	0.86, 1.77	0.263	1.15	0.77, 1.72	0.504	1.82	1.18, 2.80	0.007
13	0.86	0.59, 1.23	0.403	1.58	1.07, 2.34	0.022	1.81	1.17, 2.79	0.007
14	0.96	0.66, 1.40	0.846	1.32	0.89, 1.95	0.170	1.88	1.24, 2.86	0.003
*Age group (years)*									
< 5	1			1			1		
5–15	1.02	0.82, 1.26	0.891	1.07	0.80, 1.44	0.651	1.67	1.11, 2.50	0.013
> 15	1.07	0.87, 1.33	0.515	1.25	0.93, 1.68	0.140	1.90	1.27, 2.85	0.002
*Gender*									
Female	1			1			1		
Male	1.05	0.93, 1.19	0.400	0.97	0.83, 1.14	0.716	0.99	0.80, 1.23	0.961
*Year*									
2018	1			1			1		
2019	1.06	0.91, 1.23	0.456	1.72	1.41, 2.10	< 0.001	1.73	1.34, 2.24	< 0.001
*Body mass index (kg/m*^*2*^*)*									
< 25	1			1			1		
≥ 25	1.02	0.84, 1.24	0.838	1.57	1.22, 2.02	< 0.001	1.78	1.27, 2.49	< 0.001

*MDA* Mass drug administration

The mosquitocidal effect of IVM at day 7 post-MDA did not vary by age, gender, year of MDA and BMI (Table 5). Nevertheless, at days 14 and 21 post-treatment, the effect was significantly higher in 2019 than in 2018. Mosquito mortality was also higher at days 14 (incidence rate ratio [IRR]: 1.57; 95% CI: 1.22, 2.02; *P* < 0.001) and 21 (IRR: 1.78; 95% CI: 1.27; 2.49; *P* < 0.001) post-treatment among individuals with a BMI of at least 25 than in those with a lower BMI. The mosquitocidal effect at day 21 post-treatment was also significantly higher in older children (IRR: 1.67; 95% CI: 1.11, 2.50; *P *= 0.013) and adults (IRR: 1.90; 95% CI: 1.27, 2.85; *P* = 0.002) than in children aged < 5 years.

## Discussion

We report here the entomological effect of an intervention that aimed to reduce the human reservoir of infection using dihydroartemisinin-piperaquine and vector survival and density using IVM. While both the incidence of clinical malaria and the prevalence of infection were significantly lower in the intervention villages than in the control villages [30], the effect of IVM on the vector was less evident, with some apparently contradictory results.

Vector parity, the primary entomological endpoint, was similar between the intervention and control groups when estimated by both the CDC-LT and HLC collections, although when estimated used HLC collections parity tended to be lower in the intervention group, both in 2018 and in 2019. Parity could be determined in only 15–20% of mosquitoes captured in CDC-LT, and mostly in those captured in the early hours of the morning as those captured earlier may have died and dried up or been damaged, making dissection impossible. Conversely, mosquitoes captured in HLC can remain alive until the following morning when ovary dissection can be carried out. However, in the present study, HLC could be conducted in only a small number of villages. We therefore did not achieve the number of observations for parity we intended, reducing the study power to detect a statistically significant difference. A similar lack of effect of MDA with IVM on vector parity was observed in Burkina Faso, although serological reactivity to an anopheles salivary gland protein was significantly lower in the intervention group than in the control group, suggesting a lower exposure of individuals to mosquito bites [38]. Another potential explanation for the lack of effect on vector parity may be spillover from neighboring villages, despite a 2-km buffer zone around intervention villages, as mosquitoes can fly for a distance of up to 3 km when unfed, 9 km when sugar fed and 10 km when blood fed [39], as has been observed previously in The Gambia [40].

In 2018, vector density determined on CDC-LT collections tended to be lower in the intervention than control villages, but the difference was not statistically different; in 2019 this difference became statistically significant, possibly reflecting the higher coverage achieved in 2019. However, vector density determined on HLC collections did not differ between the intervention and control groups, although there was a tendency in 2019 for it to be higher in the intervention villages, but without reaching statistical significance. This apparent discrepancy between CDC-LT and HLC data may be due to the low number of villages sampled using HLC (8 from a total of 32 study villages), which limited the power to detect a possible difference. Considering the marked heterogeneity in entomological parameters between villages, which reflected both actual differences and the variability within the entomological collection methods [41], the tendency for a higher vector density in intervention villages observed in 2019 could be due to differences that were present prior to the intervention.

The effect of IVM on vector survival is shown by the DMFA results, obtained by feeding colony mosquitoes with blood samples from inhabitants of one intervention and one control village, respectively. Previous studies have reported a > 90% mortality of different anophelines fed on human blood collected individuals immediately after IVM treatment, with a subsequent rapidly declining efficacy over time [42]. Nevertheless, mosquito survival was found to decrease significantly for at least 28 days after feeding on blood collected from individuals after treatment with IVM at either 300 or 600 μg/kg per day for 3 days [43]. It is likely that IVM metabolites contribute to the observed mortality [44], and this should be further investigated [45]. In our study, the mosquitocidal effect of IVM was predictably stronger at 7 days after the first dose, although the effect remained detectable at 14 and 21 days after treatment [43].

Our findings on the daily survivorship allowed a detailed examination of the kinetics of mosquito mortality. Mosquitoes fed on treated blood continued to experience increased mortality up to 10–14 days after feeding, particularly when feeding on blood collected 21 days post-treatment. Such a delayed mortality could partly explain the lower-than-expected effect on vector parity as the reproductive cycles in *An. gambiae* could be as short as 2 days [40], and thus the female could lay eggs and become parous before IVM has a mosquitocidal effect. It is unclear to which extent IVM would alter vector behavior and the reproductive cycle. In Tanzania, blood digestion in mosquitoes fed on IVM-treated cattle was much slower and egg production decreased up to 15 days post-feeding [46]. Moreover, *An. arabiensis* fed on blood from IVM-treated individuals (7 and 10 days post-treatment) produced significantly fewer eggs than those fed on untreated controls [47]. Therefore, IVM may significantly alter the mosquito reproductive cycle although a subset of the mosquitoes exposed to IVM can successfully complete the gonotrophic cycle, hence diluting any effect IVM may have on the age structure of the vector populations. Nevertheless, the effect of IVM on vectors’ reproductive cycle would, by slowing down blood digestion as well as egg laying and hatching, translate into lower vector density, as observed in this trial.

Interestingly, the mosquitocidal effect of IVM, as determined in the feeding assays, was associated with host characteristics. Although in a previous study the results on blood collected at day 7 post-treatment did not show any variation by age, year of intervention and BMI [29], blood samples collected at day 14 and 21 post-treatment, when the effect of IVM was waning, did show some important differences that may be related to the pharmacokinetics of IVM. In that study, the effect of IVM was still visible at day 21 post-treatment in older children and adults but not in children aged < 5 years. Considering that one of the inclusion criteria for treatment with IVM was a body weight ≥ 15 kg or height ≥ 90 cm, this young age group included mainly children aged 4 years [48]. Nevertheless, results suggest that IVM may be more rapidly eliminated in these children than in older children and adults. Recent pharmacokinetics analyses indicate that IVM-treated children aged < 12 years reach half the peak concentration and total exposure as adults [49]. In addition, a relative underdosing in children for other drugs used in malaria control has been reported [50, 51].

In individuals with a BMI ≥ 25, IVM had a much longer and significant effect at days 14 and 21 post-treatment than in “thinner” individuals, and this effect remained apparent after controlling for age, year of intervention and gender. This effect is possibly explained by the accumulation of IVM in fat tissue, which would be released slowly, increasing the concentration of IVM in the blood over time and thus resulting in a higher and prolonged mosquitocidal effect [43]. Interestingly, a previous study showed that despite a predicted higher concentration of IVM in capillary blood, which mosquitoes probe naturally rather than venous blood, vector mortality after direct skin feeding was similar to that after membrane feeding [52].

This study has a number of limitations. In addition to the relatively low coverage achieved in 2018, a major limitation is the lack of baseline entomological data from all study villages. Due to limited resources, HLC could be implemented in only a few study villages, limiting the capacity of estimating the vector parity rates, which was the primary entomological endpoint and an important parameter to disentangle the effect of IVM from that of dihydroartemisinin-piperaquine. It was also not possible to estimate the human blood index, which limited our capacity to determine the proportion of vectors not feeding on humans and thus not exposed to the intervention.

## Conclusions

Mass drug administration efforts with IVM decreased vector density and EIR while the effect on vector parity is less clear. The individual contribution of IVM to the observed reduction in transmission cannot be clearly defined as the intervention combined IVM with dihydroartemisinin-piperaquine. A more extensive entomological evaluation of the impact of MDA with IVM alone is needed, and ongoing studies will hopefully provide such information.

## Supplementary Information

**Additional file 1:** **Table S1.** Weather information of the study area (2018 and 2019 rainy seasons).

## Data Availability

After publication, trial data will be made available on reasonable request to the corresponding author. A proposal with a detailed description of study objectives and a statistical analysis plan is needed for assessment of requests. Additional materials may also be required during the process. Deidentified participant data will be provided after approval by the sponsor and trial management group.

## Abbreviations

BMI: Body mass index
CDC-LT: US Center for Disease Control and Prevention’s light trap
CI: Confidence interval
CSP: Circum sporozoite protein
DMFA: Direct membrane feeding assay
DP: Dihydroartemisinin-piperaquine
EIR: Entomological inoculation rate
HLC: Human landing catch
IRR: Incidence rate ratio
IVM: Ivermectin
MDA: Mass drug administration
MRC: Medical Research Council
OR: Odds ratio
RR: Relative risk

## References

[1] Lines J, Kleinschmidt I (2013). Combining malaria vector control interventions: some trial design issues. Pathog Glob Health.

[2] Committee WHOMPA, Secretariat. Malaria Policy Advisory Committee to the WHO: conclusions and recommendations of eighth biannual meeting (September 2015). Malar J. 2016;15:117. 10.1186/s12936-016-1169-x. 10.1186/s12936-016-1169-x PMC4766637 26911803

[3] Ceesay SJ, Casals-Pascual C, Erskine J, Anya SE, Duah NO, Fulford AJ (2008). Changes in malaria indices between 1999 and 2007 in The Gambia: a retrospective analysis. Lancet.

[4] Ceesay SJ, Casals-Pascual C, Nwakanma DC, Walther M, Gomez-Escobar N, Fulford AJ (2010). Continued decline of malaria in The Gambia with implications for elimination. PLoS ONE.

[5] Betson M, Jawara M, Awolola TS (2009). Status of insecticide susceptibility in Anopheles gambiae s l from malaria surveillance sites in The Gambia. Malar J.

[6] Tangena JA, Adiamoh M, D'Alessandro U, Jarju L, Jawara M, Jeffries D (2013). Alternative treatments for indoor residual spraying for malaria control in a village with pyrethroid- and DDT-resistant vectors in The Gambia. PLoS ONE.

[7] Opondo KO, Jawara M, Cham S, Jatta E, Jarju L, Camara M (2019). Status of insecticide resistance in Anopheles gambiae (s.l.) of The Gambia. Parasit Vectors.

[8] Watts N, Adger WN, Ayeb-Karlsson S, Bai Y, Byass P, Campbell-Lendrum D (2017). The Lancet Countdown: tracking progress on health and climate change. Lancet.

[9] Committee WHOMPA, Secretariat. Malaria Policy Advisory Committee to the WHO: conclusions and recommendations of fifth biannual meeting (March 2014). Malar J. 2014;13:253. 10.1186/1475-2875-13-253. 10.1186/1475-2875-13-253 PMC4113024 24992998

[10] Kenea O, Balkew M, Tekie H, Gebre-Michael T, Deressa W, Loha E (2016). Human-biting activities of Anopheles species in south-central Ethiopia. Parasit Vectors.

[11] Russell TL, Beebe NW, Bugoro H, Apairamo A, Chow WK, Cooper RD (2016). Frequent blood feeding enables insecticide-treated nets to reduce transmission by mosquitoes that bite predominately outdoors. Malar J..

[12] Gatton ML, Chitnis N, Churcher T, Donnelly MJ, Ghani AC, Godfray HC (2013). The importance of mosquito behavioural adaptations to malaria control in Africa. Evolution.

[13] Ferguson HM, Dornhaus A, Beeche A, Borgemeister C, Gottlieb M, Mulla MS (2010). Ecology: a prerequisite for malaria elimination and eradication. PLoS Med..

[14] Hamid-Adiamoh M, Nwakanma D, Assogba BS, Ndiath MO, D'Alessandro U, Afrane YA (2021). Influence of insecticide resistance on the biting and resting preferences of malaria vectors in The Gambia. PLoS ONE..

[15] Williams YA, Tusting LS, Hocini S, Graves PM, Killeen GF, Kleinschmidt I (2018). Expanding the vector control toolbox for malaria elimination: a systematic review of the evidence. Adv Parasitol.

[16] Hooper PJ, Bradley MH, Biswas G, Ottesen EA (2009). The global programme to eliminate lymphatic filariasis: health impact during its first 8 years (2000–2007). Ann Trop Med Parasitol.

[17] Wolstenholme AJ, Rogers AT (2005). Glutamate-gated chloride channels and the mode of action of the avermectin/milbemycin anthelmintics. Parasitology..

[18] Kobylinski KC, Foy BD, Richardson JH (2012). Ivermectin inhibits the sporogony of Plasmodium falciparum in Anopheles gambiae. Malar J.

[19] Chaccour CJ, Kobylinski KC, Bassat Q, Bousema T, Drakeley C, Alonso P (2013). Ivermectin to reduce malaria transmission: a research agenda for a promising new tool for elimination. Malar J..

[20] Reddy MR, Overgaard HJ, Abaga S, Reddy VP, Caccone A, Kiszewski AE (2011). Outdoor host seeking behaviour of Anopheles gambiae mosquitoes following initiation of malaria vector control on Bioko Island Equatorial Guinea. Malar J.

[21] Russell TL, Govella NJ, Azizi S, Drakeley CJ, Kachur SP, Killeen GF (2011). Increased proportions of outdoor feeding among residual malaria vector populations following increased use of insecticide-treated nets in rural Tanzania. Malar J.

[22] Moiroux N, Damien GB, Egrot M, Djenontin A, Chandre F, Corbel V (2014). Human exposure to early morning Anopheles funestus biting behavior and personal protection provided by long-lasting insecticidal nets. PLoS One.

[23] Cooke MK, Kahindi SC, Oriango RM, Owaga C, Ayoma E, Mabuka D (2015). A bite before bed: exposure to malaria vectors outside the times of net use in the highlands of Western Kenya. Malar J.

[24] Ranson H, N'Guessan R, Lines J, Moiroux N, Nkuni Z, Corbel V (2011). Pyrethroid resistance in African anopheline mosquitoes: what are the implications for malaria control?. Trends Parasitol.

[25] Killeen GF (2014). Characterizing, controlling and eliminating residual malaria transmission. Malar J.

[26] Kobylinski KC, Sylla M, Chapman PL, Sarr MD, Foy BD (2011). Ivermectin mass drug administration to humans disrupts malaria parasite transmission in Senegalese villages. Am J Trop Med Hyg.

[27] Alout H, Krajacich BJ, Meyers JI, Grubaugh ND, Brackney DE, Kobylinski KC (2014). Evaluation of ivermectin mass drug administration for malaria transmission control across different West African environments. Malar J.

[28] Chaccour CJ, Rabinovich NR, Slater H, Canavati SE, Bousema T, Lacerda M (2015). Establishment of the Ivermectin Research for Malaria Elimination Network: updating the research agenda. Malar J.

[29] Ouedraogo AL, Bastiaens GJ, Tiono AB, Guelbeogo WM, Kobylinski KC, Ouedraogo A (2015). Efficacy and safety of the mosquitocidal drug ivermectin to prevent malaria transmission after treatment: a double-blind, randomized, clinical trial. Clin Infect Dis.

[30] Dabira ED, Soumare HM, Conteh B, Ceesay F, Ndiath MO, Bradley J (2022). Mass drug administration of ivermectin and dihydroartemisinin-piperaquine against malaria in settings with high coverage of standard control interventions: a cluster-randomised controlled trial in The Gambia. Lancet Infect Dis.

[31] Mwesigwa J, Slater H, Bradley J, Saidy B, Ceesay F, Whittaker C (2019). Field performance of the malaria highly sensitive rapid diagnostic test in a setting of varying malaria transmission. Malar J.

[32] Mwesigwa J, Achan J, Di Tanna GL, Affara M, Jawara M, Worwui A (2017). Residual malaria transmission dynamics varies across The Gambia despite high coverage of control interventions. PLoS One.

[33] Mboera LE, Kihonda J, Braks MA, Knols BG (1998). Short report: influence of centers for disease control light trap position, relative to a human-baited bed net, on catches of Anopheles gambiae and Culex quinquefasciatus in Tanzania. Am J Trop Med Hyg.

[34] Detinova TS, Bertram DS, World Health Organization. Age-grouping methods in Diptera of medical importance with special reference to some vectors of malaria/T.S. Detinova; [‎with]‎ an Annex on the ovary and ovarioles of mosquitos (‎with glossary)‎ by D.S. Bertram. World Health Organization monograph series no. 47. https://apps.who.int/iris/handle/10665/41724. Accessed 18 June 2018. 13885800

[35] Wirtz RA, Burkot TR, Graves PM, Andre RG (1987). Field evaluation of enzyme-linked immunosorbent assays for Plasmodium falciparum and Plasmodium vivax sporozoites in mosquitoes (Diptera: Culicidae) from Papua New Guinea. J Med Entomol.

[36] Fanello C, Santolamazza F, della Torre A (2002). Simultaneous identification of species and molecular forms of the Anopheles gambiae complex by PCR-RFLP. Med Vet Entomol.

[37] Bennett AJ, Lesch KP, Heils A, Long JC, Lorenz JG, Shoaf SE (2002). Early experience and serotonin transporter gene variation interact to influence primate CNS function. Mol Psychiatry.

[38] Foy BD, Alout H, Seaman JA, Rao S, Magalhaes T, Wade M (2019). Efficacy and risk of harms of repeat ivermectin mass drug administrations for control of malaria (RIMDAMAL): a cluster-randomised trial. Lancet.

[39] Kaufmann C, Briegel H (2004). Flight performance of the malaria vectors Anopheles gambiae and Anopheles atroparvus. J Vector Ecol.

[40] Quinones ML, Lines JD, Thomson MC, Jawara M, Morris J, Greenwood BM (1997). Anopheles gambiae gonotrophic cycle duration, biting and exiting behaviour unaffected by permethrin-impregnated bednets in The Gambia. Med Vet Entomol.

[41] Farlow R, Russell TL, Burkot TR (2020). Nextgen vector surveillance tools: sensitive, specific, cost-effective and epidemiologically relevant. Malar J.

[42] Jones JW, Meisch MV, Meek CL, Bivin WS (1992). Lethal effects of ivermectin on Anopheles quadrimaculatus. J Am Mosq Control Assoc.

[43] Smit MR, Ochomo EO, Aljayyoussi G, Kwambai TK, Abong'o BO, Chen T (2018). Safety and mosquitocidal efficacy of high-dose ivermectin when co-administered with dihydroartemisinin-piperaquine in kenyan adults with uncomplicated malaria (IVERMAL): a randomised, double-blind, placebo-controlled trial. Lancet Infect Dis.

[44] Kobylinski KC, Jittamala P, Hanboonkunupakarn B, Pukrittayakamee S, Pantuwatana K, Phasomkusolsil S (2020). Safety, pharmacokinetics, and mosquito-lethal effects of ivermectin in combination with dihydroartemisinin-piperaquine and primaquine in healthy adult thai subjects. Clin Pharmacol Ther.

[45] Tipthara P, Kobylinski KC, Godejohann M, Hanboonkunupakarn B, Roth A, Adams JH (2021). Identification of the metabolites of ivermectin in humans. Pharmacol Res Perspect.

[46] Lyimo IN, Kessy ST, Mbina KF, Daraja AA, Mnyone LL (2017). Ivermectin-treated cattle reduces blood digestion, egg production and survival of a free-living population of Anopheles arabiensis under semi-field condition in South-Eastern Tanzania. Malar J.

[47] Mekuriaw W, Balkew M, Messenger LA, Yewhalaw D, Woyessa A, Massebo F (2019). The effect of ivermectin((R)) on fertility, fecundity and mortality of Anopheles arabiensis fed on treated men in Ethiopia. Malar J.

[48] Dabira ED, Soumare HM, Lindsay SW, Conteh B, Ceesay F, Bradley J (2020). Mass drug administration with high-dose ivermectin and dihydroartemisinin-piperaquine for malaria elimination in an area of low transmission with high coverage of malaria control interventions: protocol for the MASSIV Cluster Randomized Clinical Trial. JMIR Res Protoc..

[49] Schulz JD, Coulibaly JT, Schindler C, Wimmersberger D, Keiser J (2019). Pharmacokinetics of ascending doses of ivermectin in Trichuris trichiura-infected children aged 2–12 years. J Antimicrob Chemother.

[50] WorldWide Antimalarial Resistance Network D.P.S.G. (2013). The effect of dosing regimens on the antimalarial efficacy of dihydroartemisinin-piperaquine: a pooled analysis of individual patient data. PLoS Med.

[51] Goncalves BP, Pett H, Tiono AB, Murry D, Sirima SB, Niemi M (2017). Age, weight, and CYP2D6 genotype are major determinants of primaquine pharmacokinetics in African children. Antimicrob Agents Chemother.

[52] Smit MR, Ochomo EO, Aljayyoussi G, Kwambai TK, Abong'o BO, Bousema T (2019). Human direct skin feeding versus membrane feeding to assess the mosquitocidal efficacy of high-dose ivermectin (IVERMAL Trial). Clin Infect Dis.

