# Supplementary material for: Entomological impact of mass administration of ivermectin and dihydroartemisinin-piperaquine in The Gambia: a cluster-randomized controlled trial
Source: Parasit Vectors. 2022 Nov 17;15:435. doi: 10.1186/s13071-022-05557-4 (PMC9673448; doi:10.1186/s13071-022-05557-4)
Supplement: Supplementary file 1 — Additional file 1: Table S1. Weather information of the study area (2018 and 2019 rainy seasons). [file 13071_2022_5557_MOESM1_ESM.docx]

Table S1: Weather information of the study area (2018 and 2019 rainy seasons)

| Parameters | Year | June | July | August | September | October | November | December |
| --- | --- | --- | --- | --- | --- | --- | --- | --- |
| Rainfall |  |  |  |  |  |  |  |  |
|  | 2018 | 50.5 | 200.9 | 354.5 | 321.0 | 66.3 | 0.7 | 0 |
|  | 2019 | 5.2 | 86.7 | 343.8 | 357 | 76.9 | 0 | 0 |
| Temperature |  |  |  |  |  |  |  |  |
|  | 2018 | 41.1 | 33.1 | 31.8 | 32.3 | 34.9 | 35.3 | 36.0 |
|  | 2019 | 39.2 | 33.3 | 32.8 | 32.1 | 33.8 | 36.4 | 36.4 |
| Relative humidity |  |  |  |  |  |  |  |  |
|  |  |  |  |  |  |  |  |  |
|  | 2018 | 57 | 75 | 82 | 83 | 77 | 65 | 54 |
|  | 2019 | 41 | 74 | 83 | 83 | 79 | 65 | 56 |
